# Resident Perspective on Feedback and Barriers for Use as an Educational Tool

**DOI:** 10.7759/cureus.4633

**Published:** 2019-05-10

**Authors:** Stephen Albano, Syed A Quadri, Mudassir Farooqui, Luis Arangua, Thomas Clark, Glenn M Fischberg, Emilio C Tayag, Javed Siddiqi

**Affiliations:** 1 Neurosurgery, Desert Regional Medical Center, Palm Springs, USA; 2 Neurosurgery, California Institute of Neurosciences, Thousand Oaks, USA; 3 Neurology, University of New Mexico, Albuquerque, USA; 4 Neurology, Desert Regional Medical Center, Palm Springs, USA; 5 Neurology and Neurosurgery, Desert Regional Medical Center, Palm Springs, USA

**Keywords:** residency, feedback, education

## Abstract

Background

Feedback in physician graduate medical education is not clearly defined. Some parties may view questioning as a form of feedback, others the conversations over lunch, some the comments in the operating room (OR), and still others the written evaluation at planned meetings. The lack of clarity in defining what constitutes feedback is concerning when this is considered a fundamental means of education to enhance practices and care for patients. If residents do not recognize they are receiving feedback, or the response to feedback is met with opposition, then feedback as an educational device can be limited. For this manuscript, feedback is defined as written or verbal comments regarding medical knowledge, performance, technique, or patient care.

Objective

This study attempts to identify barriers to feedback by identifying attitudes toward feedback processes through a questionnaire.

Methods

Ten questions were provided to residents at a single institution representing, emergency medicine, family medicine, internal medicine, neurology, and neurosurgery during the 2017-2018 academic year. Response was voluntary and the study was granted exemption by local institutional review board since no identifying information was collected to link responses to specific residents. Questions were formulated to identify how positive or negative a resident felt toward specific aspects of feedback.

Results

Of the possible 84 resident respondents, 40 residents participated reflecting a response of approximately 48%. Questionnaires revealed that 22.5% of respondents found feedback to be a stressful event. Sixty-seven point five percent (67.5%) of resident respondents associated the prompt that they are about to receive feedback as concerning. Only 2.5% of residents identified a meeting with the program director as a sign that the resident may be doing well. Appointments for feedback were viewed as a positive event in 12.5% of respondents. Ninety-five percent (95%) of residents do not feel that all feedback will affect their permanent record. Ten percent (10%) of residents identified receiving feedback as a positive event. Ninety-five percent (95%) of residents indicated that they have actively tried to change behavior or practices based on feedback. Forty percent (40%) of residents found themselves censoring “negative” feedback.

Conclusions

Barriers to feedback include the inability to present sensitive subjects in a constructive manner and superficial relationships between the evaluator and resident physician. Research directed at addressing these barriers could lead to improved use of feedback as an educational tool.

## Introduction

Feedback in residency is generally identified as essential for learning [1]. Feedback has many purposes; the most prominent is to encourage improvement. For this manuscript, feedback is defined as written or verbal comments regarding medical knowledge, performance, technique, or patient care. Question banks provide immediate written comments regarding medical knowledge on concepts being tested, allowing participants to identify weaknesses and improve gaps in knowledge. The written and verbal comments regarding resident performance is not always as clear as question bank comments. One of the problems is that, sometimes, residents don’t even know they are receiving feedback. There is a discrepancy between the perceptions of attending physicians providing feedback and resident physicians receiving feedback [2-5]. The lack of obviousness in actionable commentary may contribute to the feeling that residents do not feel they are receiving adequate feedback [6-7]. Feedback as an educational tool is further complicated by the quality and framing of comments. The quality or content of feedback is often sacrificed to avoid negative comments [1,3,7-12]. Feedback is further hindered by barriers such as time restraints, limited interaction between the parties involved, or training in how to convey comments, which often lead to generic or non-actionable comments [6,13]. The quality of feedback leads to mixed reactions toward the utility [7,14-16]. Regardless of the problems with feedback, the primary goal is to encourage learning and residents reported changes in behavior resulting from criticisms [17]. Therefore, this study aims to identify barriers toward the use of written and verbal comments as an educational tool by identifying resident attitudes toward feedback through a questionnaire.

## Materials and methods

A paper survey was distributed to residents from the emergency medicine, family medicine, internal medicine, neurology, and neurosurgery programs during mandatory didactics session during the 2017 academic year. A cover sheet explaining the anonymity and voluntary nature of the survey was provided with additional information for points of contact if there were any questions about the survey or if anyone wanted to view the internal review board approval certificate. After completion of the survey, all forms were collected and results tabulated as a percentage of residents responding in the affirmative. Due to the involvement of physician residents, the institutional review board reviewed the study and granted an exemption since no identifying information was being collected that could link responses to residents. After completion of the survey, all forms were collected and results reviewed. Each question response was scored from -1 to 1 based on attitude toward feedback with negative values indicating a fearful or derogatory connotation while positive values indicated a constructive or desirable connotation. Participant responses to all questions were averaged to provide a score from -1 to 1, with 1 being a positive response to feedback and -1 being a negative response to feedback.

## Results

Of the possible 84 residents practicing at the institution, 40 returned surveys, reflecting approximately 48% participation. The site did not include all specialties, but all specialties hosted at the site were surveyed. Programs consisted of emergency medicine, family medicine, internal medicine, neurology, and neurosurgery. Most of the surveys were completed by emergency medicine residents, reflecting 32.5% of participants, and post-graduate year (PGY) 1 residents, reflecting 37.5% of the respondents. A breakdown of respondents based on program in alphabetical order is shown in Table 1, and a breakdown based on PGY level is shown in Table 2.

**Table 1:** Responses Based on Program

Program	Percent of response pool
Emergency Medicine	32.5%
Family Medicine	12.5%
Internal Medicine	12.5%
Neurology	22.5%
Neurosurgery	20%

**Table 2:** Responses Based on Post-Graduate Year (PGY)

Post Graduate Year	Percent of response pool
PGY-1	37.5%
PGY-2	35%
PGY-3	25%
PGY-4	2.5%

**Figure 1:**
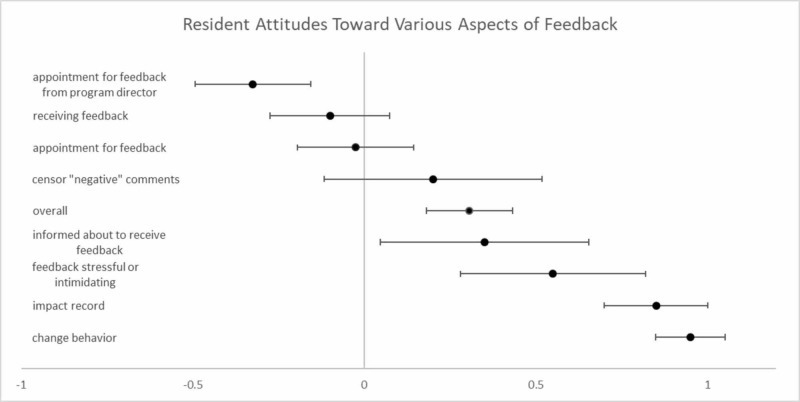
Resident Attitudes Toward Various Aspects of Feedback Average resident response to questionnaire. Zero indicates a neutral position regarding corresponding question, -1 indicates a negative position, and +1 indicates a positive position. Note: censoring “negative” comments was scored as -1 and lack of censoring was scored as +1. Error bars indicate 95% confidence interval.

Questionnaire results are summarized in Table 3 and Table 4. Table 3 indicates the percentage of respondents who replied in the affirmative and Table 4 demonstrates the average response of all participants with corresponding 95% confidence intervals, with pictorial representation in Figure 1. Questionnaires indicated many residents identified feedback as intimidating or stressful (22.5%). Along those lines, 97.5% of residents did not interpret a meeting for feedback from a program director as good news and 32.5% acknowledged feeling concerned when informed that they would be receiving feedback from the program director or faculty member. Only 12.5% identified an appointment for feedback as positive, with a minority (5%) believing that feedback would have a negative impact on record. Negative feedback was not always expressed, with 40% of residents indicating that when in the role of evaluator, they censored or withheld negative comments. Despite the prior results, 97.5% of resident respondents indicated changing their behavior or practices based on feedback.

**Table 3:** Resident Responses in the Affirmative to Various Aspects of Feedback

Item	Percent response in the affirmative
Changed behavior or practice based on feedback	97.5%
Censor “negative” feedback	40%
Concerned when informed going to be receiving feedback from program director or faculty member	32.5%
Feedback identified as intimidating or stressful	22.5%
Appointment for feedback is positive	12.5%
Receiving feedback is interpreted as positive	10%
Feedback has a negative impact on record	5%
Feedback from program director indicates good news	2.5%

**Table 4:** Average Response Score and 95% Confidence Intervals for Individual Questions

Question	Average (95% confidence interval)
Appointment for feedback from program director	-0.33 (-0.49 to -0.16)
Receiving feedback	-0.10 (-0.27 to 0.07)
Appointment for feedback	-0.03 (-0.19 to 0.14)
Censor “negative” comments	0.20 (-0.12 to 0.52)
Overall	0.31 (0.18 to 0.43)
Informed about to receive feedback	0.35 (0.05 to 0.65)
Feedback stressful or intimidating	0.55 (0.28 to 0.82)
Impact record	0.85 (0.70 to 1.00)
Change behavior	0.95 (0.85 to 1.05)

## Discussion

Most everyone will generically endorse feedback as an essential tool in education, however, many resident respondents (22.5%) indicate feedback as a stressful or intimidating event. Appointments to review this information are viewed negatively and a meeting with the program director is often not viewed in a positive light, with 32.5% of residents being concerned when they need to meet with their program director. The preconceived negative bias by a recipient toward commentary regarding performance may represent a barrier if the recipient is dismissive of comments prior to reception. As a tool, feedback is useless if not received by the party for which it is intended. Despite the aversion toward feedback, an overwhelming majority indicate a change in behavior or practice based on criticism (97.5%). The negativity may or may not be due to the feedback being disciplinary in nature. If disciplinary in nature, this could explain the adverse connotations held by residents toward criticism. The disciplinary nature could also contribute to the high number of residents changing practices based on the feedback. Therefore, if an adverse preconception toward criticism exists, then feedback as an educational tool might be limited to use in remediation. Since this is a single institution, the results are not applicable to all educational environments. However, in institutions with feedback being limited to remediation, its utility as an educational tool will also be limited to averting bad practices as opposed to inspiring and encouraging good practices. Therefore, dissociating feedback from just disciplinary discussion would allow for the broader use of criticism.

Results also indicate 40% of residents censoring “negative” feedback. Censoring includes withholding comments that may cause discomfort for the evaluator and/or the recipient. This represents a significant barrier to feedback since “negative” comments often hold the seeds for constructive criticism. Constructive criticism can be some of the most valuable feedback. However, the quality and thus value of constructive criticism is in part due to how the evaluator frames the comments and how the recipient/resident frames their interpretation. This would suggest that more training may be needed in how to professionally and tactfully handle both positive and “negative” feedback. This is concerning since physicians must communicate sensitive subjects with patients daily. Censoring feedback may also be representative of the educational culture within the program or institution. More research is needed in the quantification of educational culture and the impact of culture on educational goals.

The response to an appointment for feedback from a program director was viewed in a more negative light than from an attending faculty member. This is likely due to the opportunities for interaction. An attending faculty member who interacts with residents on a more frequent basis may be viewed with less negativity since these relationships have a chance to develop open communication and criticisms from faculty who work with a resident frequently are grounded in personal experiences. This represents another barrier to feedback - the relationship between the individuals involved. More frequent interactions do not always mean improved feedback, but at least there is an opportunity to develop shared experiences to provide more actionable feedback.

These findings are limited by the voluntary nature of the questionnaire, with a possible representative bias with those who desire feedback more willing to fill out the survey. Multiple programs were involved and the culture of feedback within each program may differ such that a subculture within a program may have been disproportionately represented in the average across all programs.

## Conclusions

Barriers to feedback include a disciplinary educational environment where feedback is only given in response to poor practices, inability to present sensitive subjects in a constructive manner, and superficial relationships between evaluator and evaluate. Research directed at addressing these barriers could lead to the improved use of feedback as an educational tool.
